# Supplementary material for: Plant Trans-Golgi Network/Early Endosome pH regulation requires Cation Chloride Cotransporter (CCC1)
Source: eLife. 2022 Jan 6;11:e70701. doi: 10.7554/eLife.70701 (PMC8791640; doi:10.7554/eLife.70701)
Supplement: Supplementary file 1. — (a) Experimental approaches taken to detect subcellular localisation of CCC1 protein. (b) Strategies to isolate a double ccc1-2 det3 or a triple ccc1-2 nhx5-2 nhx6-3 mutant [file elife-70701-supp1.docx]

Supplementary File 1a: Experimental approaches taken to detect subcellular localisation of CCC1 protein.

| **Placement** | **Type** | **Orientation** | **Promoter** | **Outcome** |
| --- | --- | --- | --- | --- |
| No tag |  |  | *CCC1* promoter | Complements *ccc1* KO |
| No tag |  |  | 2x35S | Complements *ccc1* KO |
| C-terminal | GFP | cytosolic | *CCC1* promoter | No transformants recovered (WT or *ccc1*) |
| N-terminal | GFP | cytosolic | *CCC1* promoter | No transformants recovered (WT or *ccc1*) |
| N-terminal | FLAG | cytosolic | *CCC1* promoter | No transformants recovered (WT or *ccc1*) |
| Internal, placement after amino acid residues 640, 827, 856 | GFP | cytosolic | *CCC1* promoter | No fluorescence, no complementation of *ccc1* KO |
| Internal, placement after amino acid residue 430 | mCherry | luminal | *CCC1* promoter | No fluorescence, no complementation of *ccc1* KO |
| N-terminal | GFP | cytosolic | *EXP7* | Complementation of root hair elongation in *ccc1* KO |
| No tag |  |  | *EXP7* | Complementation of root hair elongation in *ccc1* KO |
|  |  |  |  |  |

| Rabbit-derived antibody generated against CCC1 specific peptide sequence DLLVENVPRMLIVRGYHRD, no band detected at expected CCC1 size in Western blots; multiple unspecific bands detected |
| --- |

Supplementary File 1b: Strategies to isolate a double *ccc1-2 det3* or a triple *ccc1-2 nhx5-2 nhx6-3* mutant

| **Arabidopsis mutant** | **Mutant identifier** | **Zygosity** | **Gene ID** | **Reference** |
| --- | --- | --- | --- | --- |
| *ccc1-2* | SALK_145300 | Homozygous | At1g30450 | Colmenero-Flores et al. 2007 |
| *det3* | T → A mutation | Homozygous | At1g12840 | Schumacher et al. 1999 |
| *nhx5-2 nhx6-3* | GABI_094H09, SALK_145125 | Homozygous | At1g54370 & At1g79610 | Ashnest 2015 et al. 2015 |

For *det3 x ccc1-2*, homozygous single mutants were crossed with each other, no homozygous double mutants were detected; however, we were able to isolate F2 plants that were homozygous for one gene and heterozygous for the other. This confirmed that both mutant alleles had been successfully passed to the next generations following the crossing. We then genotyped the progeny of F3 plants. This was done because we assumed that any double mutants might be severely affected and we therefore expected a low frequency of these plants. Yet again, we were not able to recover a double knockout from these plants. Genotypes of F3 progeny of *det3 ccc1-2 +/-* plants was as followed: 55% *det3 ccc1-2 +/-*, and 45% *det3 ccc1-2 +/+*, 0% *det3 ccc1-2*.

For *nhx5* and *nhx6*, we used *a nhx5-2 x nhx6-3* homozygous double mutant (Ashnest et al. 2015) and crossed it with a homozygous *ccc1-2* plant. These two genes are on the same chromosome arm on chromosome 1 (AT1G54370 and AT1G79610) and rarely segregate during crosses. In addition, *CCC1* (AT1G30450) is also on the same chromosome arm very close to the centromere. Segregation of the *ccc1-2* allele and the *nhx5 nhx6* did almost not occur. We genotyped >100 F2 plants and were only able to obtain one triple mutant. This plant did not produce any seed (Fig.S7). The strong genetic linkage of the genes also prevented us from working with seeds from heterozygous plants, as the frequency of triple mutant progeny is too low.
